# Supplementary material for: Doa10/MARCH6 architecture interconnects E3 ligase activity with lipid-binding transmembrane channel to regulate SQLE
Source: Nat Commun. 2024 Jan 9;15:410. doi: 10.1038/s41467-023-44670-5 (PMC10776854; doi:10.1038/s41467-023-44670-5)
Supplement: Supplementary file 1 — Supplementary Information [file 41467_2023_44670_MOESM1_ESM.pdf]

Supplementary Fig. 1

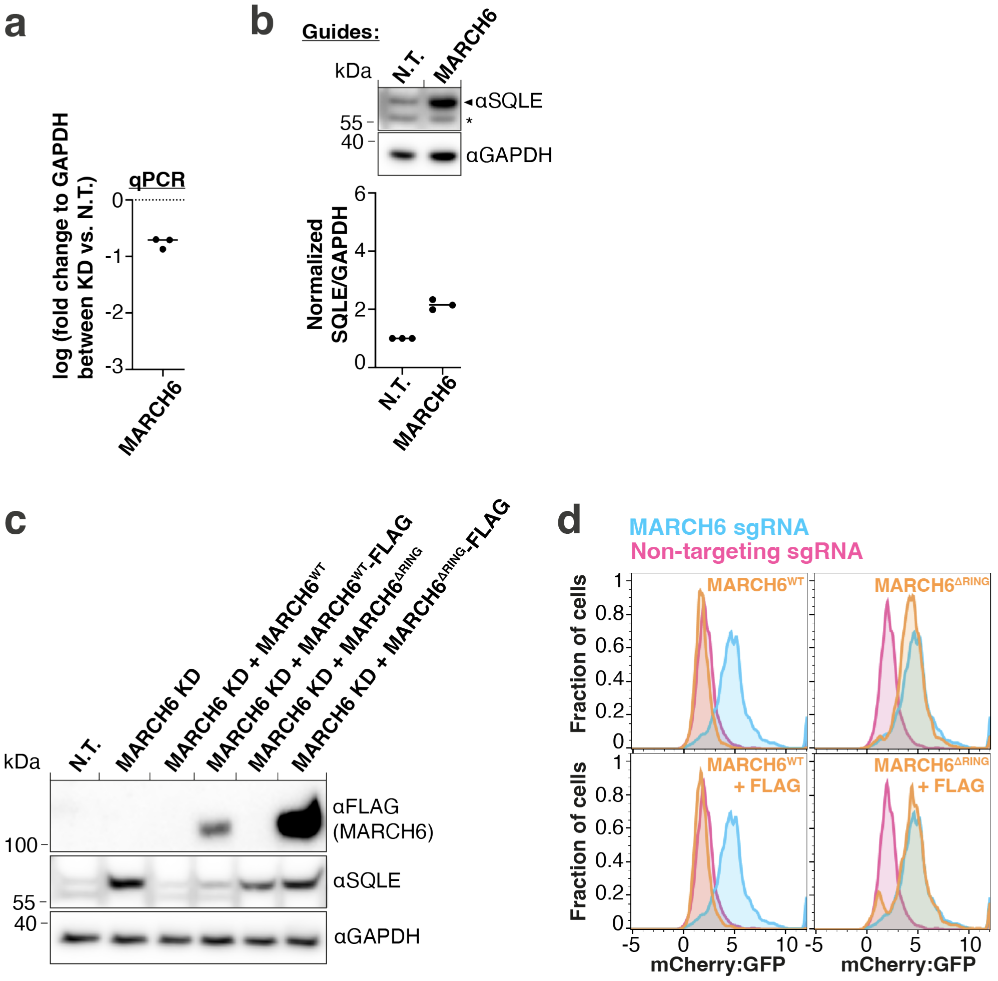

Supplementary Fig. 1: **Validating CRISPRi knock-down of endogenous MARCH6 and rescue with WT or ΔRING MARCH6.**

a) Quantitative PCR (qPCR) analysis of transcript levels in K562-dCas9-zim3 CRISPRi knock-down cells. Transcript levels for MARCH6 were measured and compared to GAPDH when expressing non-targeting (N.T.) or gene-targeting sgRNA. Data points were acquired in three biological replicates. Source data is provided in a Source Data file.

b) Endogenous immunoblot against SQLE in K562-dCas9-zim3 CRISPRi knock-down cells. The ratio of SQLE:GAPDH band intensities is plotted below for three biological replicates. Source data is provided in a Source Data file.

c) Immunoblot against endogenous SQLE and overexpressed $MARCH6^{WT}$ or $MARCH6^{\Delta RING}$. Expression of either untagged or C-terminally FLAG-tagged $MARCH6^{WT}$ in K562-dCas9-zim3 CRISPRi MARCH6 knock-down cells rescues degradation of endogenous SQLE, while this is not the case when either untagged or FLAG-tagged $MARCH6^{\Delta RING}$ is expressed. Note the strong stabilization of $MARCH6^{\Delta RING}$ levels compared to $MARCH6^{WT}$. Representative result shown from three independent biological replicates. Source data is provided in a Source Data file.

d) Flow cytometry analysis of SQLE reporter levels in $MARCH6^{WT}$ or $MARCH6^{\Delta RING}$ rescue cell lines. SQLE reporter expressing K562-dCas9-zim3 CRISPRi MARCH6 knock-down cells were rescued with untagged or FLAG-tagged $MARCH6^{WT}$ or $MARCH6^{\Delta RING}$. As a reference, SQLE reporter levels in non-targeting guide expressing cells are shown in pink. Histogram presentation of relative mCherry fluorescence normalized to GFP as an expression control. Representative result shown from three independent biological replicates.

Supplementary Fig. 2

a

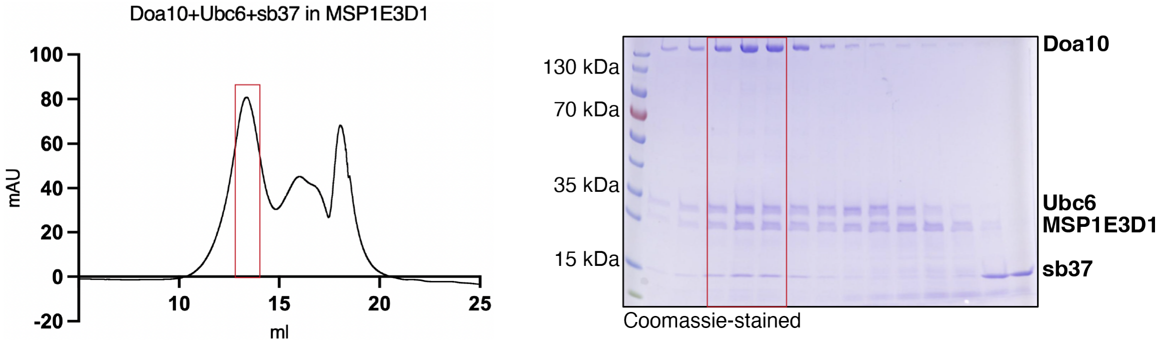

b

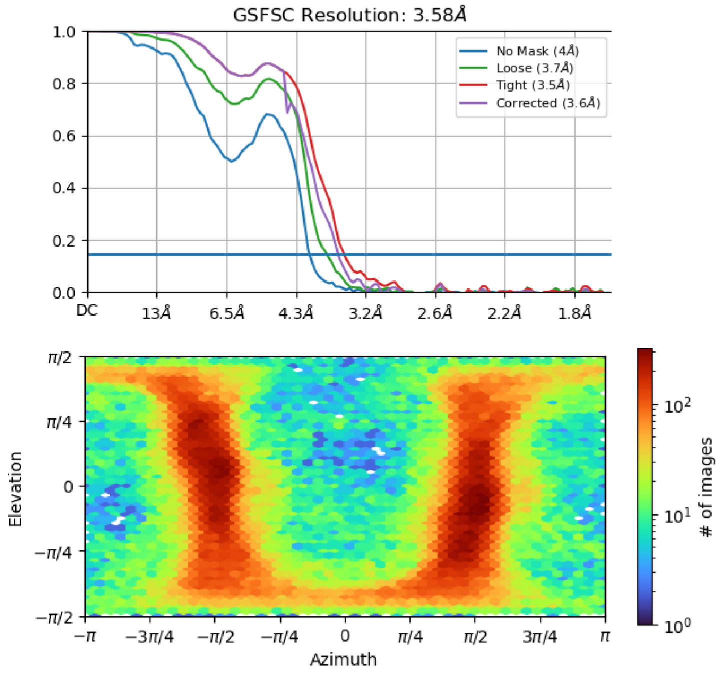

Supplementary Fig. 2 continued

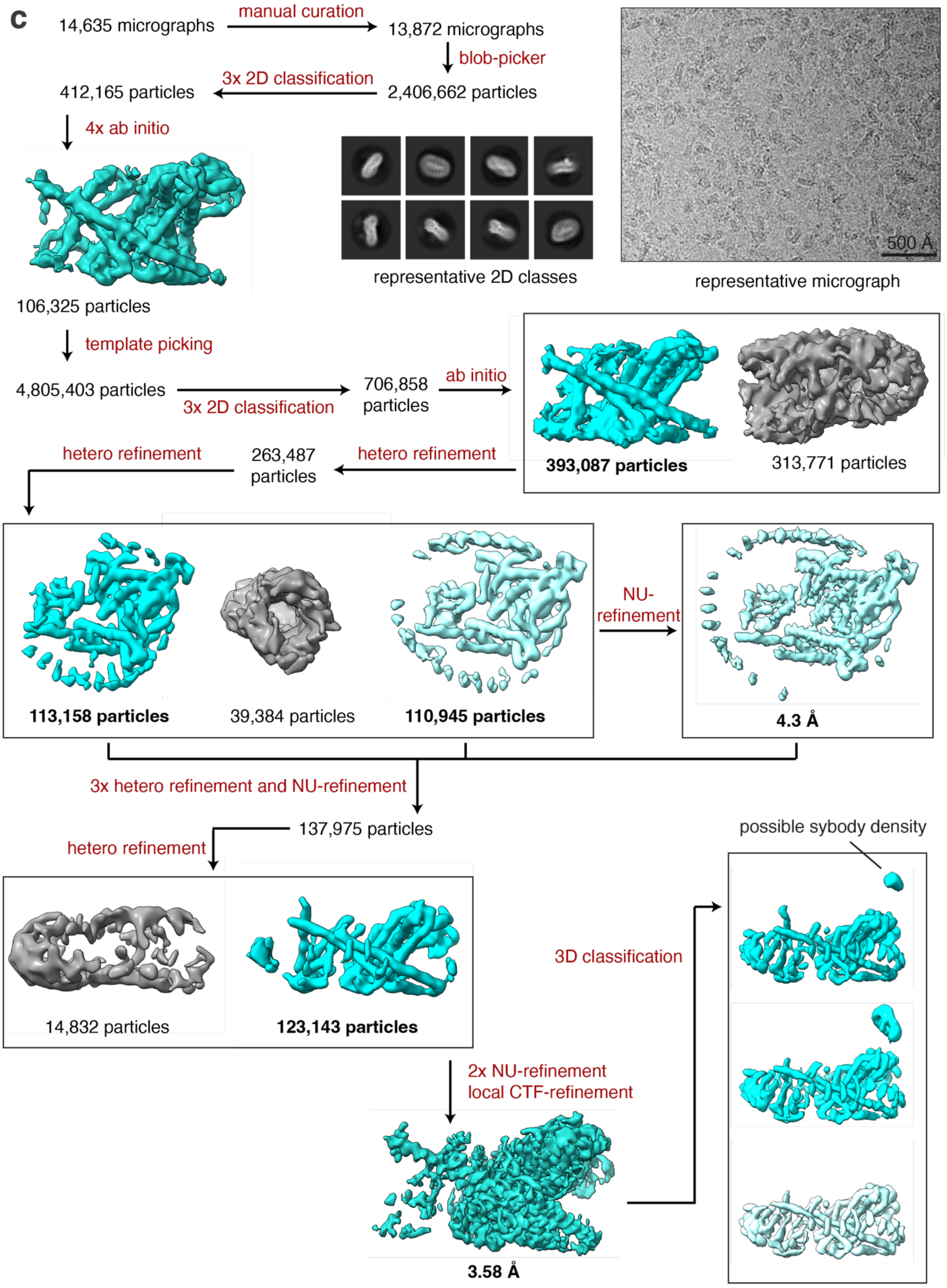

Supplementary Fig. 2: **Doa10 purification and cryo-EM data processing.**

a) *Left*: Size exclusion profile of a Superose 6 run. *Right*: Corresponding Coomassie-stained SDS-PAGE gel of the final Doa10-Ubc6-sb37 complex in MSP1E3D1. A red box indicates the fractions pooled for cryo-EM analysis.

b) *Top*: FSC curve of the final reconstruction with a global resolution of ~3.6 Å. *Bottom*: Angular distribution of the particles of the final reconstruction.

c) Processing scheme of the high-resolution cryo-EM analysis of the Doa10-Ubc6-sb37 complex in MSP1E3D1 including a representative micrograph and representative 2D classes. A final 3D classification job did not result in further refinement of classes (except for the possible low-resolution sybody density).

Supplementary Fig. 3

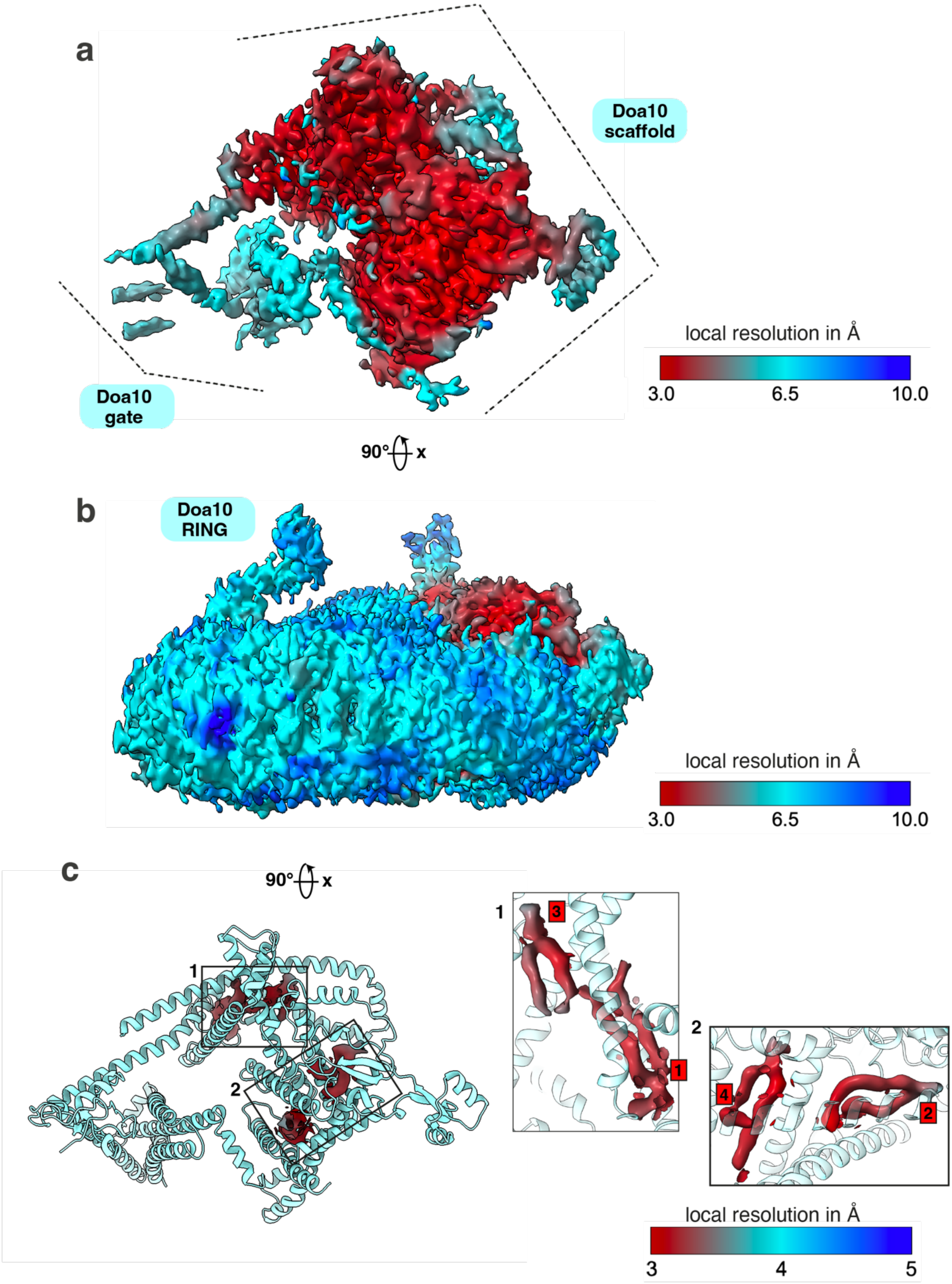

Supplementary Fig. 3: Local resolution analysis of the Doa10 cryo-EM map.

a) Local resolution map of Doa10 showing a resolution range from 3.0 Å (red) to 6.5 Å (cyan). While the scaffold domain has an average resolution of 3 Å, the flexible gate helices are resolved at a much lower resolution of 4.5 – 5 Å.

b) Local resolution map of Doa10 at higher thresholds. The nanodisc density and the RING domain towering above the membrane channel are resolved at a lower resolution of 4 – 7 Å.

c) Cartoon depiction of Doa10 with cryo-EM density shown for the four built lipids within the scaffold. The density is colored based on local resolution ranging from 3.0 Å (red) to 4 Å (cyan). The lipids are resolved at resolution < 3.5 Å.

Supplementary Fig. 4

a

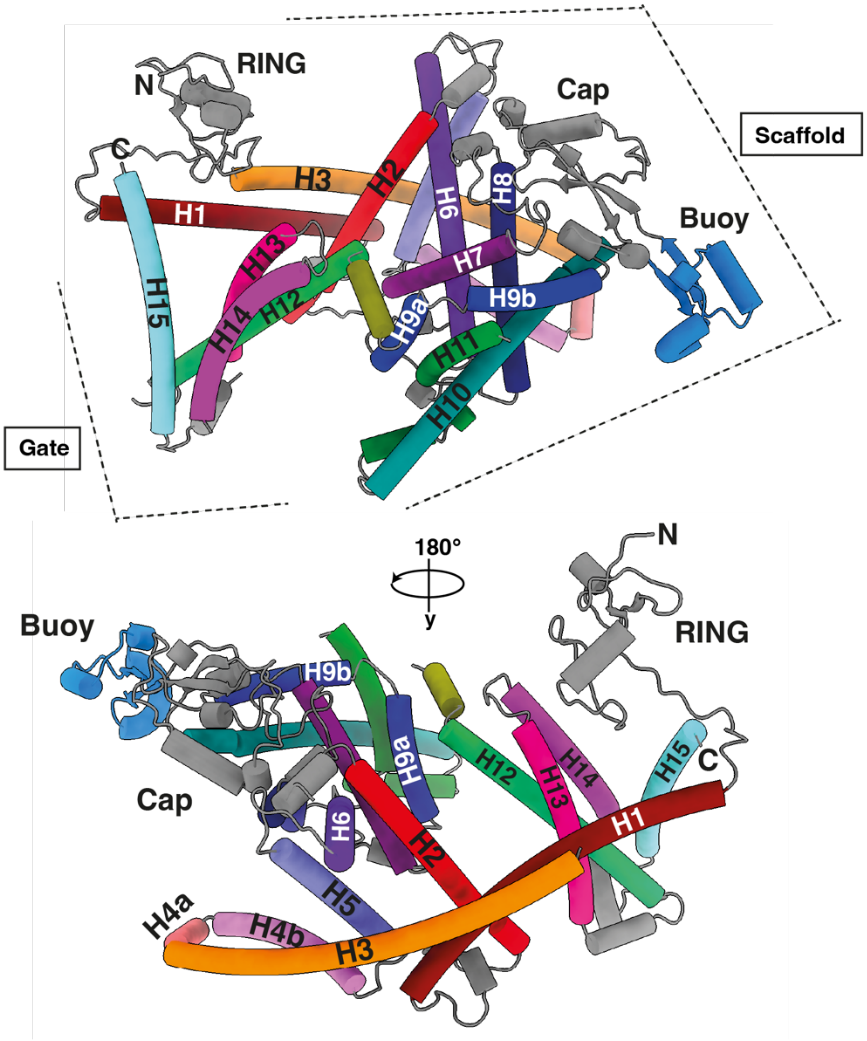

b

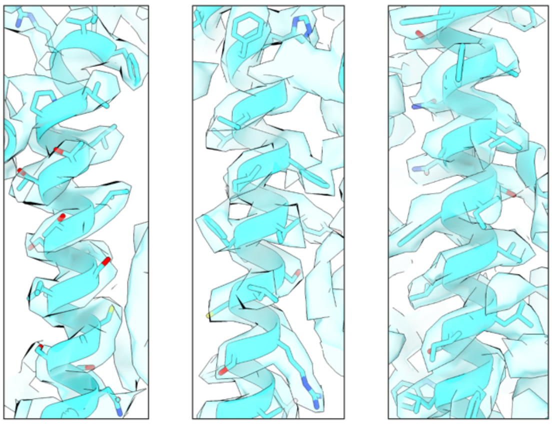

c

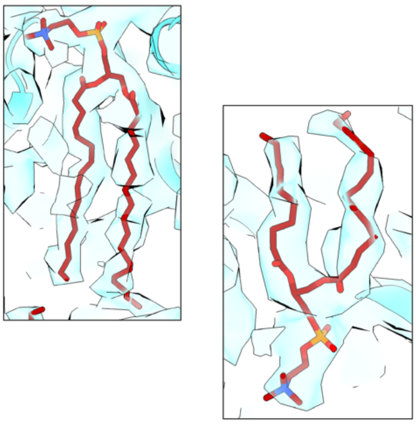

Supplementary Fig. 4: **Overview of the Doa10 cryo-EM structure with all TMH.**

a) Doa10 structure as cartoon representation. Transmembrane helices are colored in different colors and labeled as Helix 1 to Helix 15. The Buoy domain is colored in blue and the RING domain predicted by AlphaFold is included at the N-terminus (grey). The scaffold and gate domain are labeled with black dashes.

b) Well-resolved cryo-EM density for the scaffold of Doa10. Representative density for Helix 5, Helix 8 and Helix 10 in light blue (transparent) with the Doa10 structure as cartoon representation. Amino acid sidechains of the helices are shown as sticks.

c) Representative cryo-EM density for the tightly-bound lipids 1 and 3. The map is shown in light blue, while the lipids are shown in dark red as sticks.

Supplementary Fig. 5

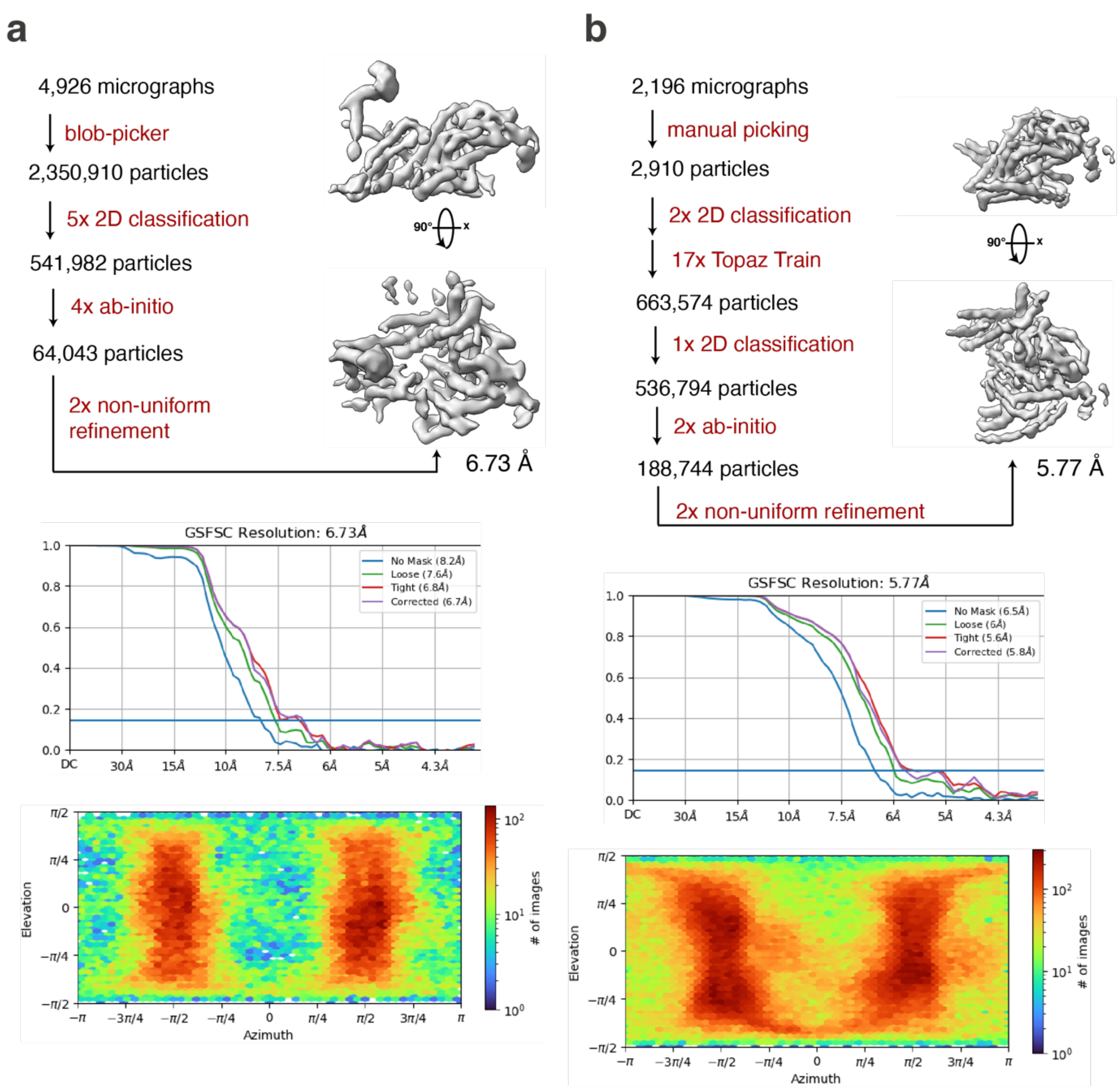

Supplementary Fig. 5: Processing schemes for the low resolution cryo-EM data sets of Doa10.

a) Processing scheme of the low-resolution cryo-EM analysis of the Doa10-Ubc6-sb37 complex in MSP1E3D1 (∅ ~12.9 nm) with FSC curve and angular distribution of the final reconstruction.

b) Processing scheme of the low-resolution cryo-EM analysis of Doa10 in the wider nanodisc MSP2N2 (∅ 15-16.5 nm) with FSC curve and angular distribution of the final reconstruction.

Supplementary Fig. 6

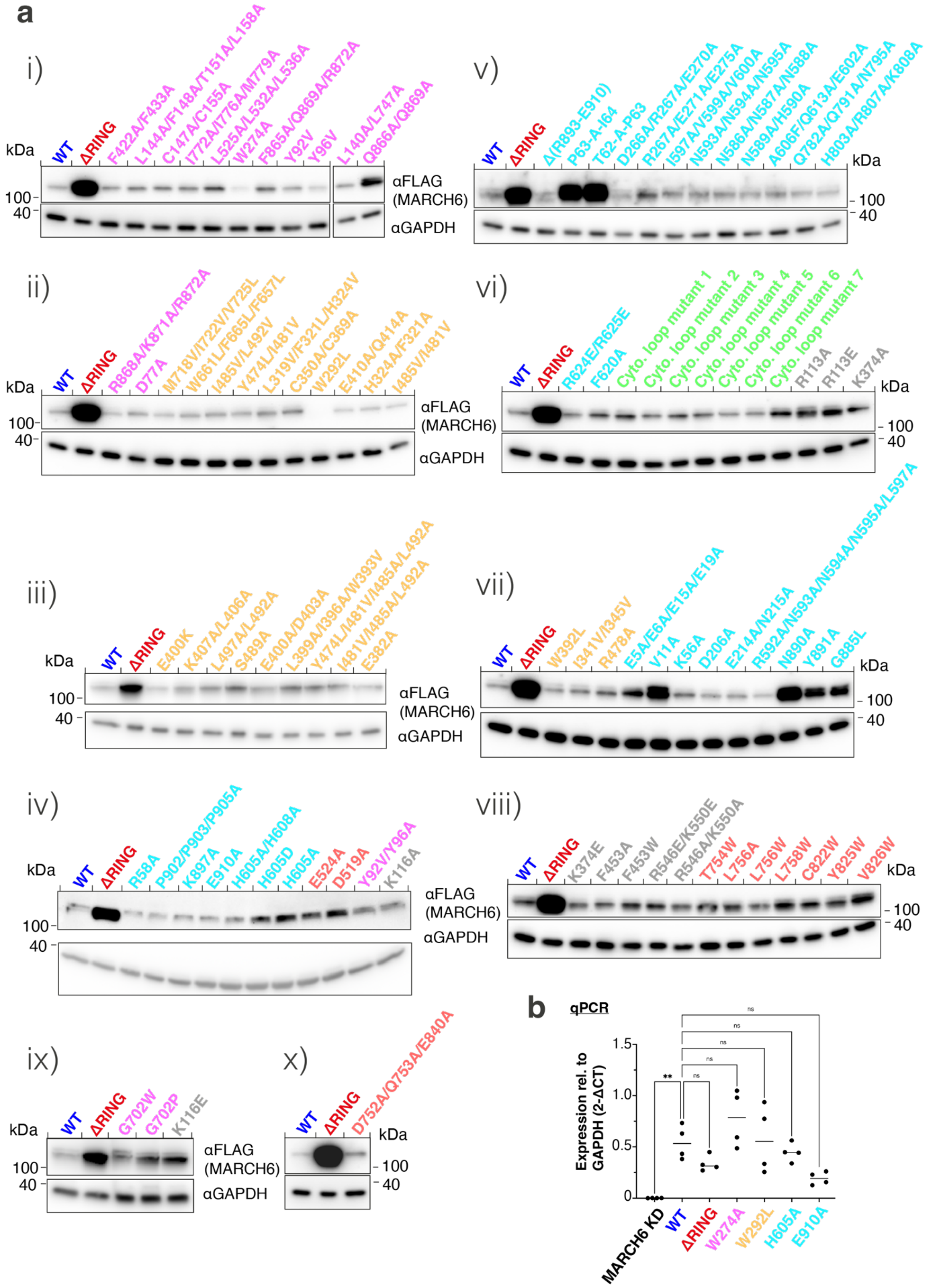

Supplementary Fig. 6: **Western blots showing protein abundance for each MARCH6 mutant.**

a) α-FLAG Western blots of every MARCH6-FLAG rescue mutant used in the SQLE reporter screen. WT MARCH6 (first lane on every gel) and MARCH6 ΔRING (second lane on every gel) are loaded as comparison. All mutants are expressed and most mutants show protein abundance similar to the WT. In addition, some mutants have a higher abundance resembling the catalytically dead ΔRING mutant (especially mutants close to the RING domain, labeled in cyan). Mutants are labeled with the same color code used for the SQLE screen categorizing their position within the structure. α-GAPDH blots are shown under every mutant as a loading control. All mutants with blot numbering are listed in the Supplementary Data. Source data is provided in a Source Data file.

b) qPCR for low abundant MARCH6 mutants. mRNA levels are depicted relative to GAPDH control levels and compared to WT MARCH6 using ANOVA. Transcript levels for MARCH6 mutants show no significant change compared to WT for four independent biological replicates. Source data is provided in a Source Data file.

Supplementary Fig. 7

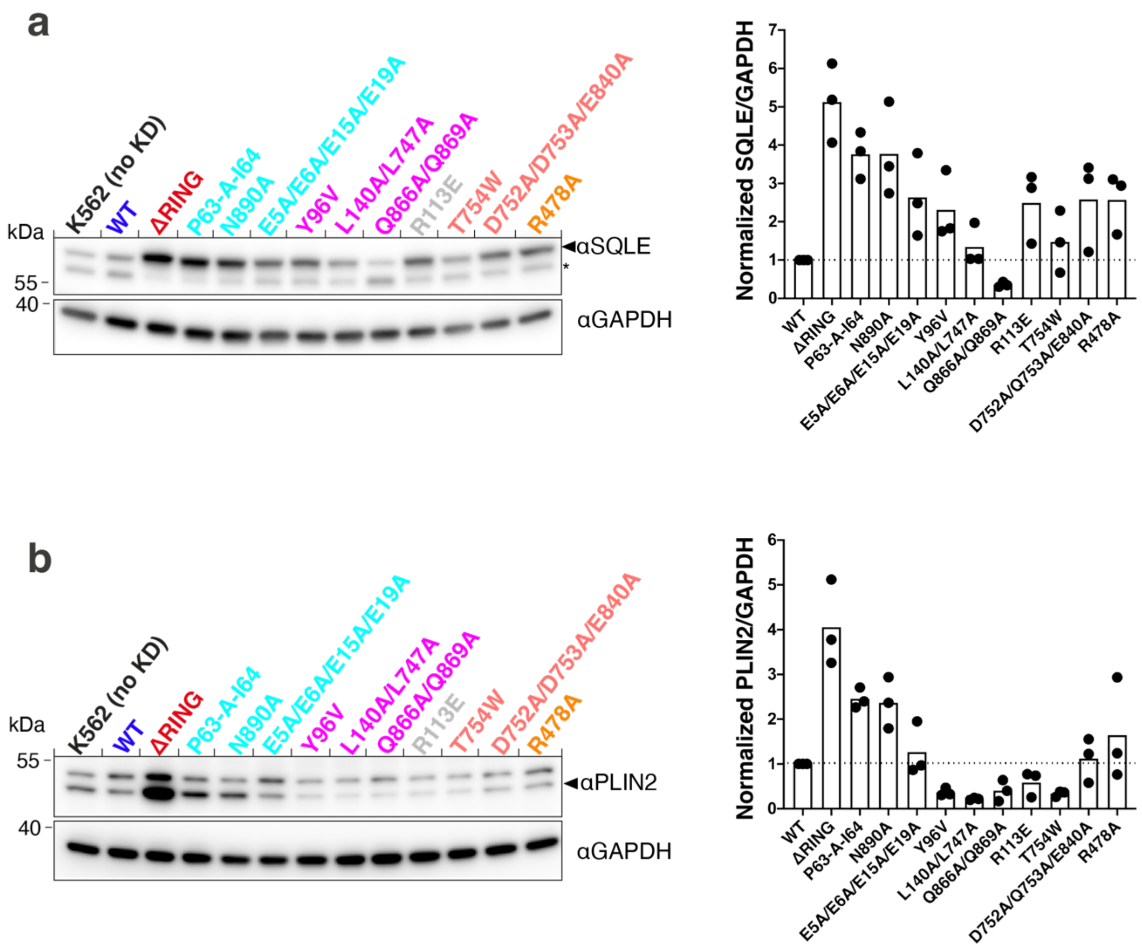

Supplementary Fig. 7: **Western blot to determine endogenous levels of SQLE and PLIN2 in a panel of mutant MARCH6 cell lines.**

Western Blots against endogenous a) SQLE and b) PLIN2. Stability of endogenous SQLE mirrors the effect of the mutants on the SQLE reporter. Similar effects are also observed for endogenous PLIN2 with exception for Y96V and R116E. On the right side, a normalized quantification of the western blots is depicted for the stability of both substrates for three independent biological replicates. Source data is provided in a Source Data file.

Supplementary Fig. 8

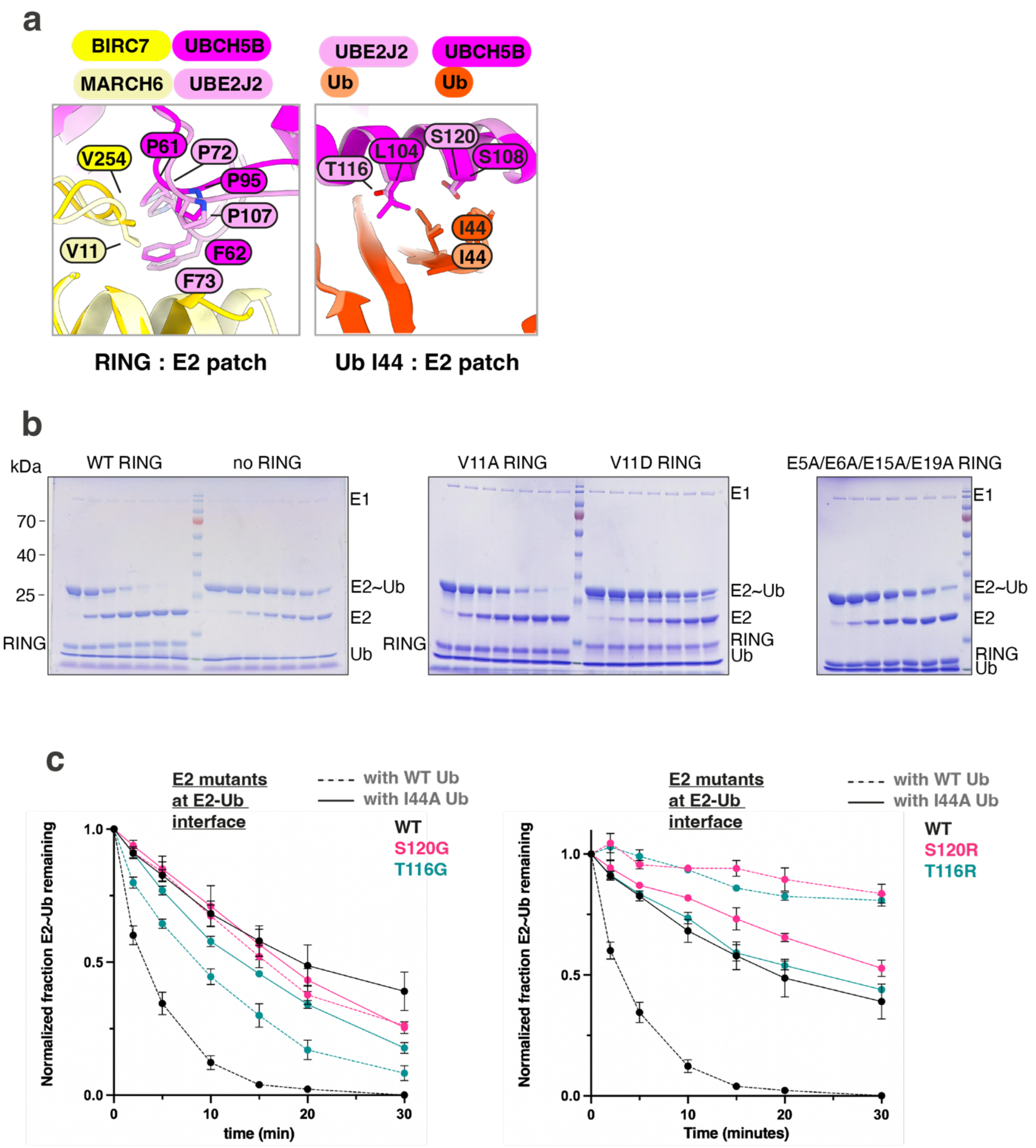

Supplementary Fig. 8 continued

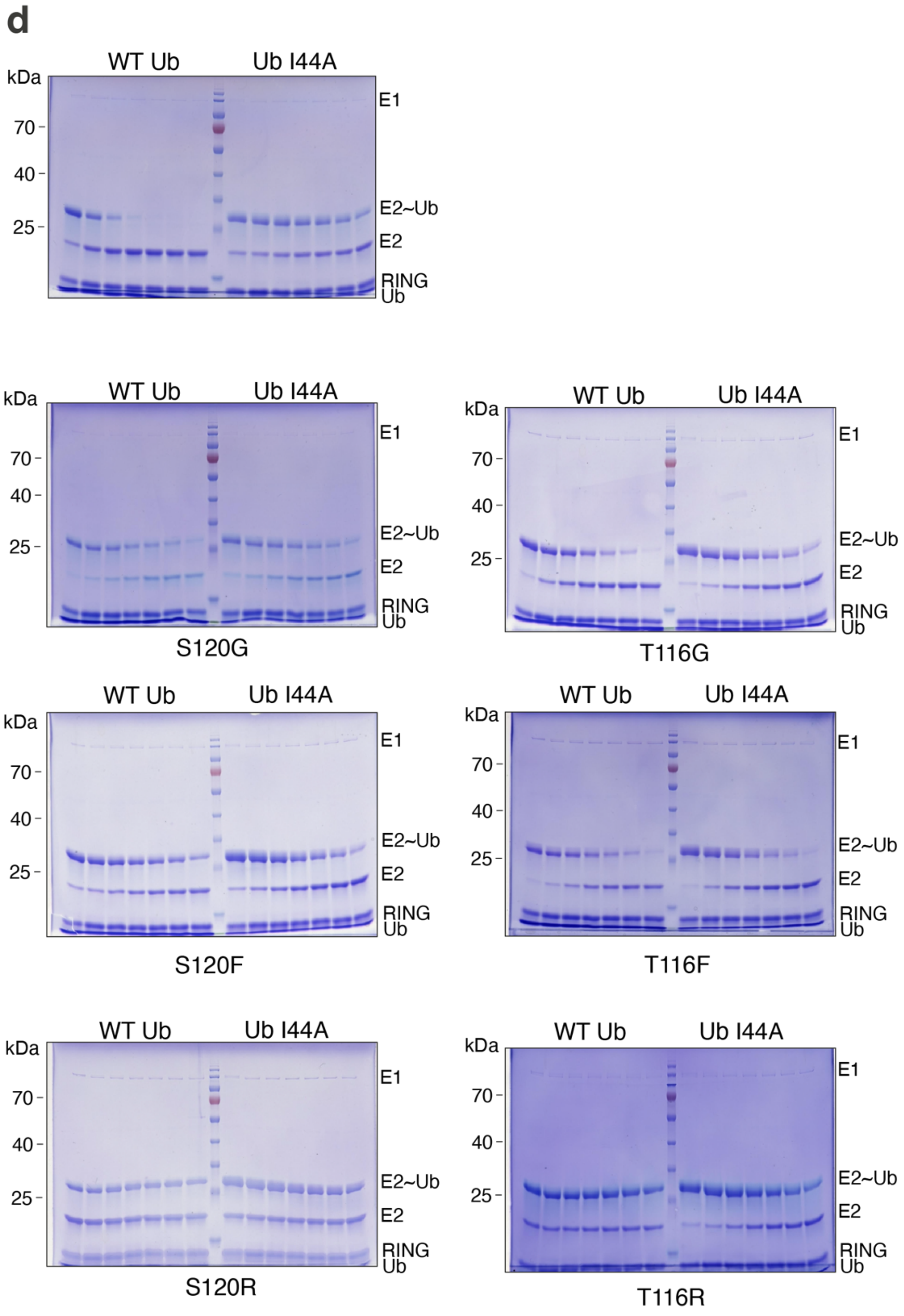

Supplementary Fig. 8: Ubiquitin discharge from UBE2J2 catalytic domain to validate AF predicted catalytic model.

a) Comparison of the AF model of UBE2J2 catalytic domain (light pink), isolated MARCH6 RING domain (light yellow) and ubiquitin (salmon) with the structure of BIRC7 RING (yellow), UbcH5b (pink) and ubiquitin (orange) (PDB: 4AUQ). *Left*: Close-up of interactions at the RING-E2 interface. Most notably a conserved valine (V11 in MARCH6), which reaches into a hydrophobic pocket formed by the E2. *Right*: Close-up of E2-ubiquitin interactions. The I44A patch of ubiquitin is aligned next to T116 and a conserved S120 of UBE2J2.

b) Representative Coomassie-stained gels depicting the discharge of ubiquitin from WT UBE2J2 dependent on MARCH6 RING domain (no RING addition, WT RING, three RING mutants). Quantification of the discharge assay can be found in Fig. 4b for three independent experiments.

c) Quantification of ubiquitin discharge from UBE2J2 depending on different UBE2J2 and ubiquitin mutants. *Left*: Mutating S120 or T116 in UBE2J2 (predicted to interact with the I44A patch of ubiquitin) to glycine decreases discharge capability only marginally (dashed line) and using I44A ubiquitin does not rescue this defect. *Right*: Mutating the respective residues to arginine results in a strong defect in discharge (dashed line) which can be partially rescued using I44A ubiquitin. Results from three independent experiments. Source data is provided in a Source Data file.

d) Representative Coomassie-stained gels depicting the discharge of WT or I44A ubiquitin from UBE2J2 catalytic domain (WT or S120F, S120G, S120R or T116F, T116G, T116R). MARCH6 WT RING domain is added to all reactions to stimulate the discharge. Quantification of the discharge assays are depicted above in c). In addition, comparing the 0 minute time points of the different E2 versions shows loading defects of the E2 with ubiquitin for S120R and T116R. Results from three independent experiments. Source data is provided in a Source Data file.

Supplementary Fig. 9

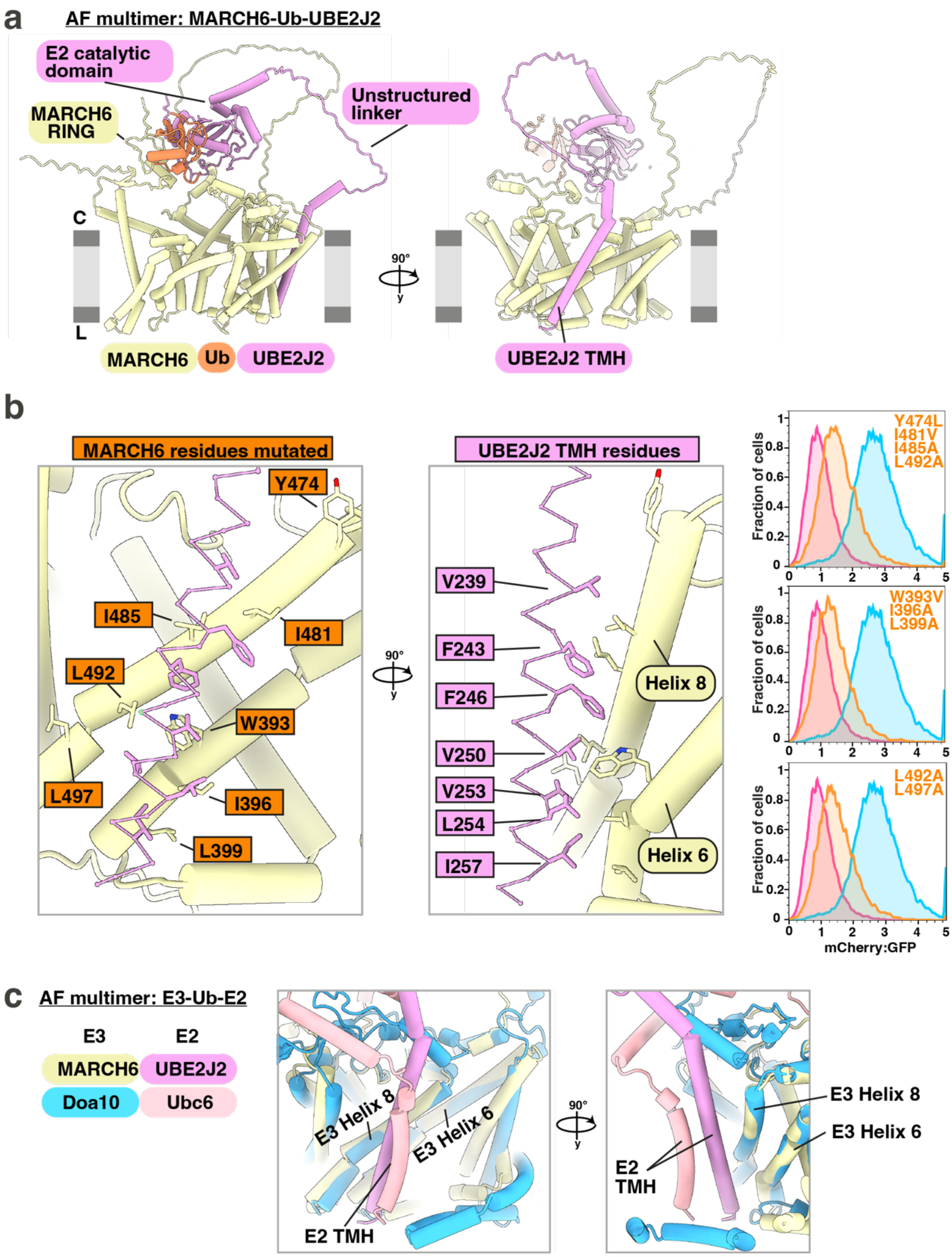

Supplementary Fig. 9: **AF prediction of the UBE2J2 binding site in MARCH6, with mutant effects shown for residues mutated at the predicted interface.**

a) AF multimer prediction for the assembly of the MARCH6 (light yellow) complex with its E2 protein UBE2J2 (pink) and ubiquitin (orange). The TMH of UBE2J2 is bound to the scaffold domain of MARCH6 and the catalytic domain is positioned next to the RING domain together with ubiquitin in the closed conformation.

b) *Left*: Close up view of the predicted interactions between the TMH of UBE2J2 (pink) and the scaffold domain of MARCH6 (light yellow), especially Helix 6 and Helix 8. Residues mutated in MARCH6 in the SQLE stability screen are highlighted on the left side, while residues in UBE2J2 predicted to form interactions with MARCH6 are labeled on the right side. *Right*: Flow cytometry panels for mutants having an effect in this region. Histogram depiction of relative mCherry fluorescence normalized to GFP as an expression control. Representative result shown from four independent biological replicates.

c) Human vs. yeast comparison of predicted AF multimer complexes focusing on the binding of the TMH of the E2 to the scaffold domain of the E3. Both E2s, UBE2J2 (pink) and Ubc6 (rose), bind to their respective E3s, MARCH6 (light yellow) and Doa10 (blue), in a similar binding site.

Supplementary Fig. 10

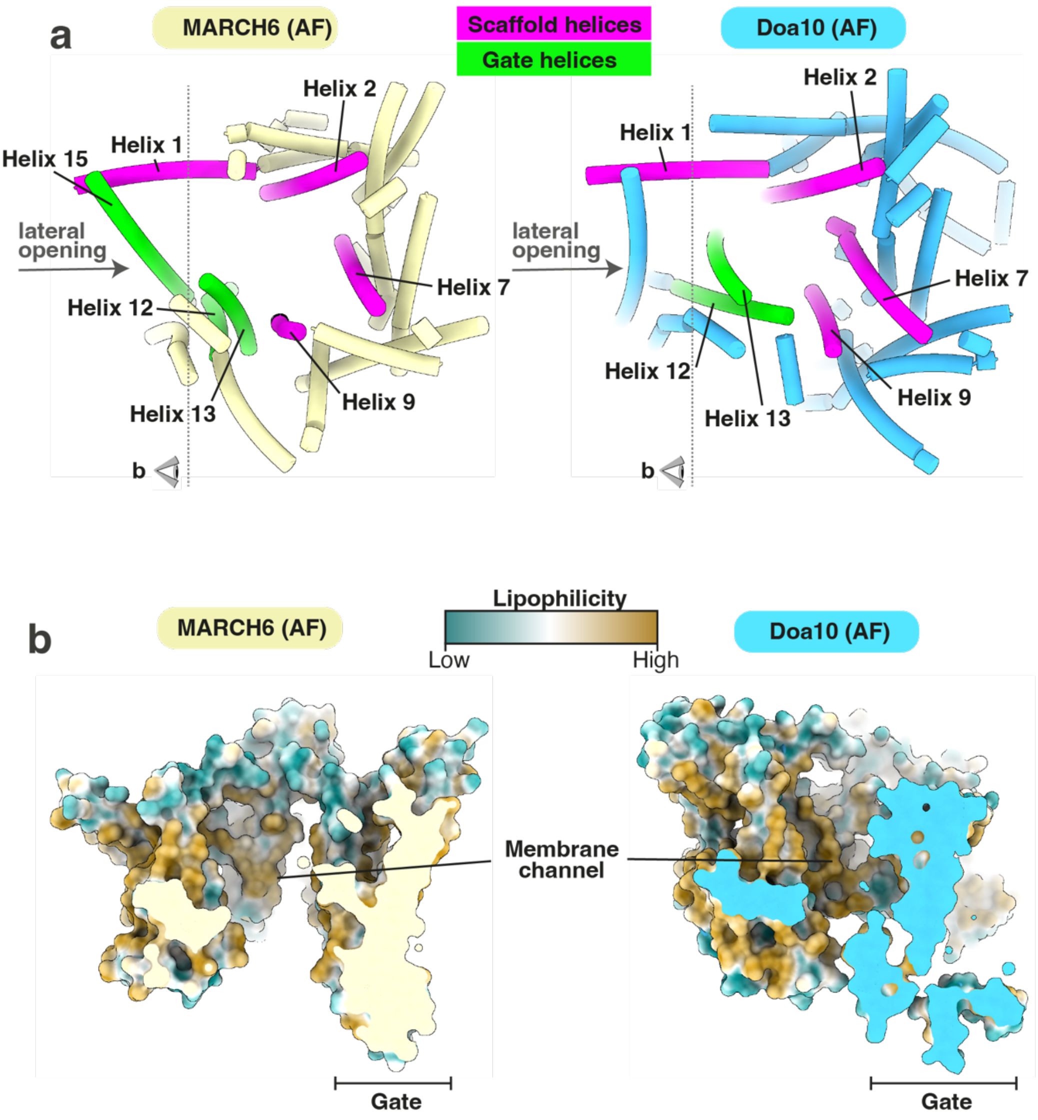

Supplementary Fig. 10: Details and properties of the MARCH6 membrane channel.

a) Top view of the AF predicted model of MARCH6 (left, light yellow) and Doa10 (right, blue). Helices lining the membrane channel are highlighted in bright pink (scaffold helices) or bright green (gate helices).

b) Surface representation of MARCH6 (left, light yellow) and Doa10 (right, blue) colored based on the lipophilicity of the residues (yellow: lipophile, blue: hydrophile). A slice through the surface in side view unveils the lipophilic interior of the membrane channel.

Supplementary Fig. 11

a

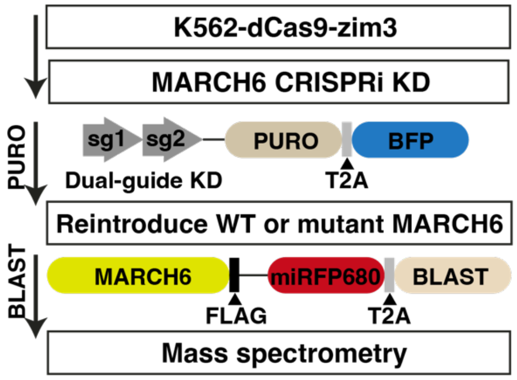

b

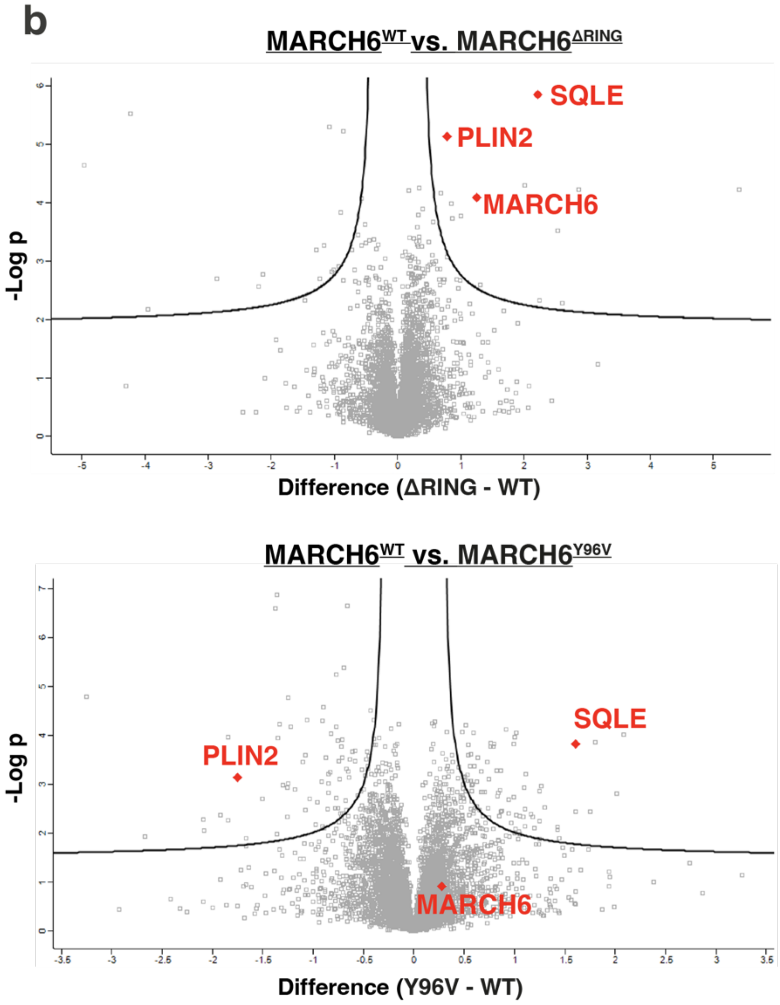

Supplementary Fig. 11: **Total proteomics of MARCH6$^{\Delta RING}$ and MARCH6^{Y96V}, a strongly defective channel lining mutant.**

a) Experimental design of the proteomics experiments. MARCH6 knockdowns are created in K562-dCas9-zim3 cell lines with two MARCH6 targeting CRISPRi guide RNAs. MARCH6 variants are reintroduced in a rescue experiment and after blasticidin selection total proteomics were measured for the sample.

b) Total proteomics for MARCH6^{WT} compared with MARCH6$^{\Delta RING}$ or MARCH6^{Y96V}. Two proteins whose levels are regulated by MARCH6, SQLE and PLIN2, as well as MARCH6 itself, are enriched in the catalytically dead ΔRING mutant. For the Y96V mutant, SQLE is stabilized compared to WT, while an opposing effect is observed for PLIN2, which is destabilized in the Y96V mutant. Notably, MARCH6 abundance is not affected by the mutant compared to WT. Experiments done for three independent biological replicates. Raw proteomics data is uploaded to the ProteomeXchange Consortium with identifier PXD047499.

Supplementary Fig. 12

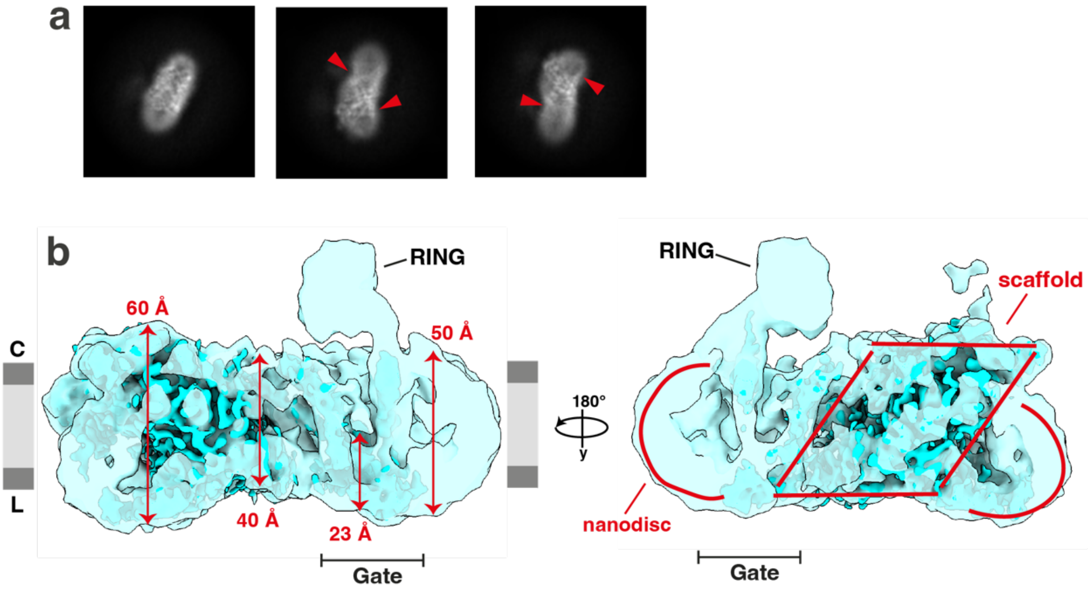

Supplementary Fig. 12: Membrane distortion and relative thinning by Doa10.

a) 2D projections of the high-resolution Doa10 cryo-EM map for visualization of the nanodisc shape. Kinks and relative thinning are highlighted with red arrows.

b) Side views of the cryo-EM map of Doa10 at two thresholds are shown. The shape of the scaffold domain is drawn on the right side (red lines), together with the outlines for the nanodisc. The wedge-shaped architecture of the scaffold domain kinks the nanodisc and distorts the parallel lipid bilayer. Furthermore, the height of the scaffold domain (60 Å) leads to relative membrane thinning within the central channel of 30-40 Å. Both, relative membrane thinning and bilayer kinking and distortion could aid membrane protein extraction.

Supplementary Fig. 13

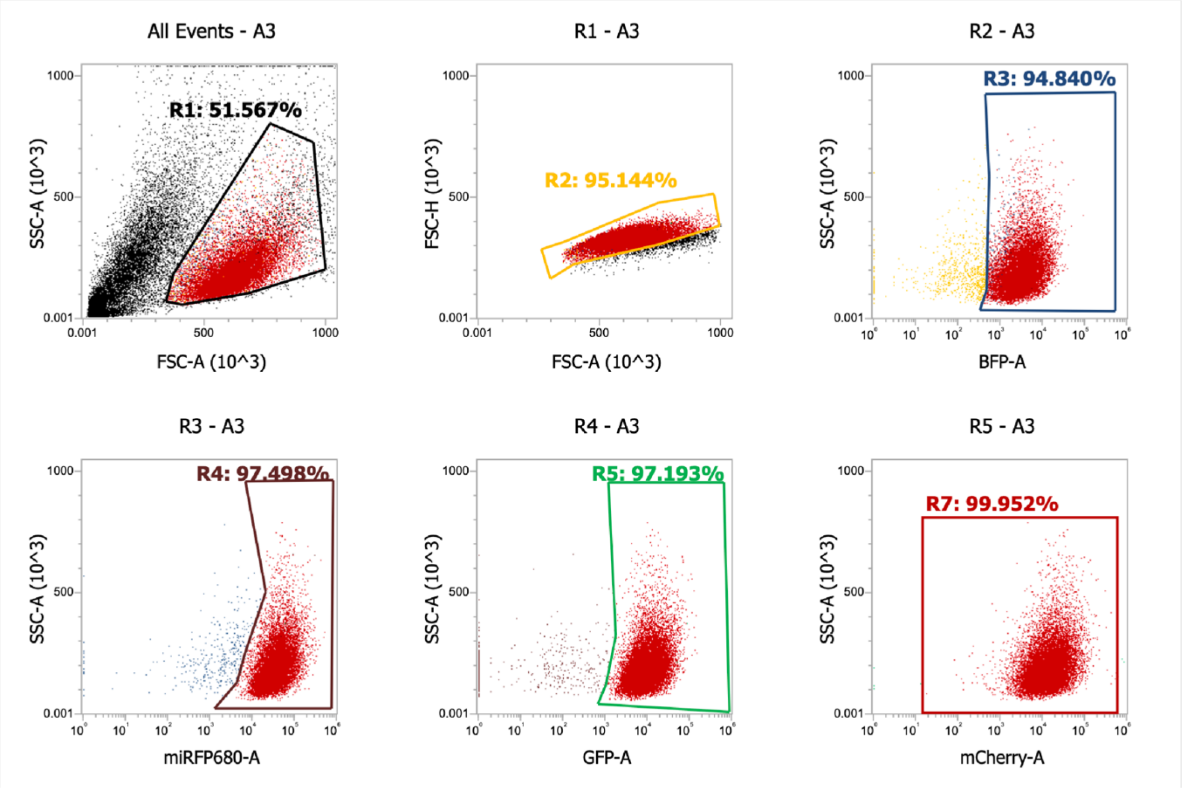

Supplementary Fig. 13: Gating strategy for the SQLE-reporter stability assay

Gating strategy for identifying single, live cells (first two gates, R1 and R2), which express CRISPRi guides for the knockdown of endogenous MARCH6 and BFP (third gate, R3) and MARCH6 rescue versions (WT or mutants) together with miRFP680 (fourth gate, R4). Finally the cells were gated for GFP expression (fifth gate, R5). Fluorescence for GFP and mCherry was measured for this subpopulation of cells.

Supplementary Table 1: Cryo-EM data collection, refinement and validation statistics

	Doa10+Ubc6+sb37 in MSP1E3D1 (EMDB-17597) (PDB: 8PDA)	Doa10+Ubc6+sb37 in MSP1E3D1 (EMDB-17609)	Doa10 in MSP2N2 (EMDB-17610)
**Data collection and processing**			
Microscope	FEI Titan Krios	Glacios	Glacios
Magnification	105,000	22,000	22,000
Voltage (kV)	300	200	200
Electron exposure (e–/Å^2)	60	70	60
Defocus range (μm)	-0.7 ~ -2.8	-1.2 ~ -3.3	-1.2 ~ -3.3
Pixel size (Å)	0.8512	1.885	1.885
Symmetry imposed	C1	C1	C1
Micrographs (no.)	14,635	4,926	2,196
Initial particle images (no.)	4,805,403	2,350,910	663,574
Final particle images (no.)	123,143	64,043	188,744
Map resolution (Å)	3.58	6.73	5.77
FSC threshold	0.143	0.143	0.143
**Refinement***			
Initial model used	Doa10 AlphaFold prediction		
Model resolution (Å)	3.58		
FSC threshold	0.143		
Model composition			
Non-hydrogen atoms	6,274 (6,736)		
Protein residues	856 (973)		
Ligands	4		
B factors (Å^2)			
Protein	39.6 (42.0)		
Ligand	16.9		
R.m.s. deviations			
Bond lengths (Å)	0.002 (0.005)		
Bond angles (°)	0.490 (0.736)		
Validation			
MolProbity score	1.1 (1.2)		
Clashscore	3.1 (3.2)		
Poor rotamers (%)	0.2 (0.4)		
Ramachandran plot			
Favored (%)	98.13 (97.26)		
Allowed (%)	1.87 (2.74)		
Disallowed (%)	0		

*Refinement values are given for the Doa10 structure with and without (in parenthesis) the RING domain. PDB for Doa10 without the RING domain: 8PD0.
