# Supplementary material for: Doa10/MARCH6 architecture interconnects E3 ligase activity with lipid-binding transmembrane channel to regulate SQLE
Source: Nat Commun. 2024 Jan 9;15:410. doi: 10.1038/s41467-023-44670-5 (PMC10776854; doi:10.1038/s41467-023-44670-5)
Supplement: Supplementary file 3 — Description of Additional Supplementary Files [file 41467_2023_44670_MOESM3_ESM.pdf]

File name: Supplementary Data 1

Description: List of all plasmids used in this study. Provided are the name, the content and the source of the plasmid, in addition to notes and the mutant number for MARCH6 variants used in the reporter assay.

File name: Supplementary Data 2

Description: List of DNA sequences for the main plasmids, as well as corresponding protein sequences of the expressed constructs.

File name: Supplementary Data 3

Description: MARCH6_mutant_effects. Summary of the SQLE reporter stability assay conducted with different MARCH6 mutants. Listed are the number of the mutant, the corresponding amino acid mutation, notes, the mean and standard error of the mean (SEM) of the median of the mCherry:GFP ratio, in addition to the number of replicates used for each mutant. *P* Values and significance summary calculated with one-way ANOVA for each mutant compared with the WT are listed next. The final column provides the western blot numbering for probing the abundance level of each mutant in Supplementary Figure 6.
