# Supplementary material for: Doa10/MARCH6 architecture interconnects E3 ligase activity with lipid-binding transmembrane channel to regulate SQLE
Source: Nat Commun. 2024 Jan 9;15:410. doi: 10.1038/s41467-023-44670-5 (PMC10776854; doi:10.1038/s41467-023-44670-5)
Supplement: Supplementary file 7 — Reporting Summary [file 41467_2023_44670_MOESM7_ESM.pdf]

nature portfolio

Corresponding author(s): Bastian Bräuning and Brenda A. Schulman

Last updated by author(s): Dec 5, 2023

Reporting Summary

Nature Portfolio wishes to improve the reproducibility of the work that we publish. This form provides structure for consistency and transparency in reporting. For further information on Nature Portfolio policies, see our Editorial Policies and the Editorial Policy Checklist.

Statistics

For all statistical analyses, confirm that the following items are present in the figure legend, table legend, main text, or Methods section.

n/a	Confirmed	
☐	☒	The exact sample size (*n*) for each experimental group/condition, given as a discrete number and unit of measurement
☐	☒	A statement on whether measurements were taken from distinct samples or whether the same sample was measured repeatedly
☐	☒	The statistical test(s) used AND whether they are one- or two-sided<br>*Only common tests should be described solely by name; describe more complex techniques in the Methods section.*
☒	☐	A description of all covariates tested
☐	☒	A description of any assumptions or corrections, such as tests of normality and adjustment for multiple comparisons
☐	☒	A full description of the statistical parameters including central tendency (e.g. means) or other basic estimates (e.g. regression coefficient) AND variation (e.g. standard deviation) or associated estimates of uncertainty (e.g. confidence intervals)
☐	☒	For null hypothesis testing, the test statistic (e.g. *F*, *t*, *r*) with confidence intervals, effect sizes, degrees of freedom and *P* value noted<br>*Give P values as exact values whenever suitable.*
☒	☐	For Bayesian analysis, information on the choice of priors and Markov chain Monte Carlo settings
☒	☐	For hierarchical and complex designs, identification of the appropriate level for tests and full reporting of outcomes
☒	☐	Estimates of effect sizes (e.g. Cohen's *d*, Pearson's *r*), indicating how they were calculated

Our web collection on statistics for biologists contains articles on many of the points above.

Software and code

Policy information about availability of computer code

Data collection	Gel/Western Blot imaging: Amersham Imager 600; Cryo-EM: SerialEM v3.8.0-b5; Flow cytometry: Attune NxT, qPCR: BioRad CFX96 Real-Time System
Data analysis	RELION v3.1.1, CryoSparc v3.1.0, Gautomatch v0.56, CTFFIND v4.1; Chimera v1.15, ChimeraX v1.4, PyMol v2.3.4; COOT v0.9.6, Phenix.refine v1.19.2, AlphaFold2, AlphaFold 2.2 multimer, FlowJo v10.8.2, ThermoFisher Attune NxT Software, Perseus v2.0.9.0., MaxQuant v2.2.0.0, BioRad CFX Manager v2.1, GraphPad Prism 10.0.2, ImageJ (2.0.0)

For manuscripts utilizing custom algorithms or software that are central to the research but not yet described in published literature, software must be made available to editors and reviewers. We strongly encourage code deposition in a community repository (e.g. GitHub). See the Nature Portfolio guidelines for submitting code & software for further information.

Data

Policy information about availability of data

All manuscripts must include a data availability statement. This statement should provide the following information, where applicable:

- Accession codes, unique identifiers, or web links for publicly available datasets
- A description of any restrictions on data availability
- For clinical datasets or third party data, please ensure that the statement adheres to our policy

The structural data will be available from EMDB and RCSB upon manuscript publication. Doa10 in MSP1E3D1 without the RING domain: EMDB-17597 [https://www.ebi.ac.uk/emdb/EMD-17597], PDB: 8PD0 [https://doi.org/10.2210/pdb8PD0/pdb]; Doa10 in MSP1E3D1 with the RING domain: PDB: 8PDA [https://

doi.org/10.2210/pdb8PDA/pdb]. Low resolution cryo-EM maps were deposited to EMDB and are available via the following accession numbers: Doa10 complex in MSP1E3D1: EMDB-17609 [https://www.ebi.ac.uk/emdb/EMD-17609], Doa10 in MSP2N2: EMDB-17610 [https://www.ebi.ac.uk/emdb/EMD-17610]. Mass spectrometry data were deposited in the ProteomeXchange Consortium with dataset identifier PXD047499. Raw gel images, western blots and raw data underlying plots are provided as Source Data or Supplementary Figures.

Research involving human participants, their data, or biological material

Policy information about studies with human participants or human data. See also policy information about sex, gender (identity/presentation), and sexual orientation and race, ethnicity and racism.

Reporting on sex and gender	No research involving human participants was performed.
Reporting on race, ethnicity, or other socially relevant groupings	No research involving human participants was performed.
Population characteristics	No research involving human participants was performed.
Recruitment	No research involving human participants was performed.
Ethics oversight	No research involving human participants was performed.

Note that full information on the approval of the study protocol must also be provided in the manuscript.

Field-specific reporting

Please select the one below that is the best fit for your research. If you are not sure, read the appropriate sections before making your selection.

- [x] Life sciences
- [ ] Behavioural & social sciences
- [ ] Ecological, evolutionary & environmental sciences

For a reference copy of the document with all sections, see nature.com/documents/nr-reporting-summary-flat.pdf

Life sciences study design

All studies must disclose on these points even when the disclosure is negative.

Sample size	Calculations to predetermine optimal sample size were not performed. Instead sample sizes were chosen to ensure significant and reliable interpretation of the results. The rationale of the sample size was based on previous experience with similar experiment types and depending on the variability of the data. Generally, we performed at least three independent experiments to ensure data reproducibility.
Data exclusions	No data was excluded.
Replication	All replications were successful and yielded reproducible results. All experiments were performed in at least three independent experiments including the respective controls. Exact number of replicates are given in the figure legends or supplementary data.
Randomization	No grouping was performed.
Blinding	No grouping was performed.

Reporting for specific materials, systems and methods

We require information from authors about some types of materials, experimental systems and methods used in many studies. Here, indicate whether each material, system or method listed is relevant to your study. If you are not sure if a list item applies to your research, read the appropriate section before selecting a response.

Materials & experimental systems

n/a	Involved in the study	
[ ]	[x]	Antibodies
[ ]	[x]	Eukaryotic cell lines
[x]	[ ]	Palaeontology and archaeology
[x]	[ ]	Animals and other organisms
[x]	[ ]	Clinical data
[x]	[ ]	Dual use research of concern
[x]	[ ]	Plants

Methods

n/a	Involved in the study	
[x]	[ ]	ChIP-seq
[ ]	[x]	Flow cytometry
[x]	[ ]	MRI-based neuroimaging

Antibodies

Antibodies used	anti-SQLE (Proteintech 12544-1-AP, rabbit, 1:1000), anti-PLIN2 (Cell Signaling 45535, rabbit, 1:500), anti-FLAG (Sigma F1804, mouse, 1:2000), anti-GAPDH (Cell Signaling D16H11, rabbit, 1:2000), anti-mouse-HRP (Cell Signaling 7076, 1:5000), anti-rabbit-HRP (Cell Signaling 7074, 1:5000)
Validation	Control western blots were run to validate specificity of all primary antibodies. All antibodies bound to their specific endogenous proteins in K562 cell lines.<br>anti-SQLE (https://www.ptglab.com/products/SQLE-Antibody-12544-1-AP.htm), was also binding the molecular weight marker<br>anti-PLIN2 (https://www.cellsignal.com/products/primary-antibodies/perilipin-2-antibody/45535)<br>anti-FLAG (https://www.sigmaaldrich.com/DE/de/product/sigma/f1804#product-documentation)<br>anti-GAPDH (https://www.cellsignal.com/products/primary-antibodies/gapdh-d16h11-xp-rabbit-mab/5174)

Eukaryotic cell lines

Policy information about cell lines and Sex and Gender in Research

Cell line source(s)	K562 UCOE-SFFV-Zim3-dCas9-P2A-hygro cell lines were a generous gift from Alina Guna, , Sf9 cells were obtained from ThermoFisher Scientific (identifier: 11496015), and Hi5 (BTI-TN-5B1-4) cells were obtained from ThermoFisher Scientific (catalog number: B85502).
Authentication	Cell lines were not authenticated.
Mycoplasma contamination	Cell lines were tested regularly for mycoplasma contamination. No contamination was observed.
Commonly misidentified lines (See ICLAC register)	No commonly misidentified cell lines were used in this study.

Plants

Seed stocks	No plant material was used for the study.
Novel plant genotypes	No plant material was used for the study.
Authentication	No plant material was used for the study.

Flow Cytometry

Plots

Confirm that:

- [x] The axis labels state the marker and fluorochrome used (e.g. CD4-FITC).
- [x] The axis scales are clearly visible. Include numbers along axes only for bottom left plot of group (a 'group' is an analysis of identical markers).
- [x] All plots are contour plots with outliers or pseudocolor plots.
- [x] A numerical value for number of cells or percentage (with statistics) is provided.

Methodology

Sample preparation	K562 UCOE-SFFV-Zim3-dCas9-P2A-hygro cell lines stably expressing a SQLE-mCherry-P2A-GFP reporter were sorted for GFP expression. Next, knockdown for MARCHF6 by CRISPRi followed by re-introduction of WT or mutant MARCH6 with a Flag-tag was performed.
Instrument	ThermoFisher Attune NxT
Software	ThermoFisher Attune NxT Software, FlowJo 10.8.2
Cell population abundance	Selection of the cells by cell sorting or resistance makers were done until the population of the desired cells were >90%

Gating strategy	Only one third of the cells with the highest GFP signal were sorted. After selection, cells were gated for being alive and singular, positive for BFP, miRFP680 and GFP/mCherry.

☒ Tick this box to confirm that a figure exemplifying the gating strategy is provided in the Supplementary Information.
